# Effects of cannabinoid drugs on the deficit of prepulse inhibition of startle in an animal model of schizophrenia: the SHR strain

**DOI:** 10.3389/fphar.2014.00010

**Published:** 2014-02-06

**Authors:** Raquel Levin, Fernanda F. Peres, Valéria Almeida, Mariana B. Calzavara, Antonio W. Zuardi, Jaime E. C. Hallak, José Alexandre S. Crippa, Vanessa C. Abílio

**Affiliations:** ^1^Department of Pharmacology, Federal University of São PauloSão Paulo, Brazil; ^2^Laboratório Interdisciplinar de Neurociãncias Clínicas, Department of Psychiatry, Federal University of São PauloSão Paulo, Brazil; ^3^Department of Neuroscience and Behavior, University of São PauloRibeirão Preto, Brazil; ^4^National Institute of Science and Technology in Translational Medicine, National Council for Scientific and Technological DevelopmentRibeirão Preto, Brazil

**Keywords:** schizophrenia, SHR, PPI, cannabinoid drugs, animal model

## Abstract

Clinical and neurobiological findings suggest that the cannabinoids and the endocannabinoid system may be implicated in the pathophysiology and treatment of schizophrenia. We described that the spontaneously hypertensive rats (SHR) strain presents a schizophrenia behavioral phenotype that is specifically attenuated by antipsychotic drugs, and potentiated by proschizophrenia manipulations. Based on these findings, we have suggested this strain as an animal model of schizophrenia. The aim of this study was to evaluate the effects of cannabinoid drugs on the deficit of prepulse inhibition (PPI) of startle, the main paradigm used to study sensorimotor gating impairment related to schizophrenia, presented by the SHR strain. The following drugs were used: (1) WIN55212,2 (cannabinoid agonist), (2) rimonabant (CB_1_ antagonist), (3) AM404 (anandamide uptake inhibitor), and (4) cannabidiol (CBD; indirect CB_1_/CB_2_ receptor antagonist, among other effects). Wistar rats (WRs) and SHRs were treated with vehicle (VEH) or different doses of WIN55212 (0.3, 1, or 3 mg/kg), rimonabant (0.75, 1.5, or 3 mg/kg), AM404 (1, 5, or 10 mg/kg), or CBD (15, 30, or 60 mg/kg). VEH-treated SHRs showed a decreased PPI when compared to WRs. This PPI deficit was reversed by 1 mg/kg WIN and 30 mg/kg CBD. Conversely, 0.75 mg/kg rimonabant decreased PPI in SHR strain, whereas AM404 did not modify it. Our results reinforce the role of the endocannabinoid system in the sensorimotor gating impairment related to schizophrenia, and point to cannabinoid drugs as potential therapeutic strategies.

## INTRODUCTION

Prepulse inhibition (PPI) of startle is characterized by the reduction of an acoustic startle reflex to an intense acoustic stimulus (pulse) when immediately preceded by a lower intensity stimulus (prepulse; Swerdlow et al., 2001). PPI is considered an operational measure of sensorimotor gating, and is extensively used in translational models of psychosis since it appears to be present in all mammals, including rats and humans (Swerdlow et al., 1994, 2000), and is disrupted in schizophrenia patients (Braff and Geyer, 1990; Braff et al., 1992, 1999; Weike et al., 2000).

The spontaneously hypertensive rat (SHR) strain was developed by selecting brother–sister mating Wistar rats (WRs) with a hypertensive phenotype (Okamoto and Aoki, 1963). Along with the hypertension presented by these animals, the inbreeding also selected behavioral abnormalities that lead to suggest them as a putative animal model of attention deficit/hyperactivity disorder (Sagvolden and Sergeant, 1998; Russell, 2007). This strain presents sustained attention problems, hyperactivity in a variety of behavioral paradigms and impulsivity (Sagvolden et al., 1992; Russell, 2007). Nevertheless, the absence of beneficial effects of psychostimulants (used to treat this disorder) on these behaviors in adult SHRs (van den Bergh et al., 2006; Bizot et al., 2007; Calzavara et al., 2009) has been described. In fact, some behavioral changes are even potentiated by these drugs (Calzavara et al., 2009). It is noteworthy that most of the studies using the SHR strain to investigate attention deficit/hyperactivity disorder were performed using the Wistar-Kyoto strain (WKY – developed by inbreeding WRs without hypertension) as controls, which may be unsuitable since it has been reported that WKY animals present an inactivity when compared to WRs (Pare, 1992, 1994), and do not show genetic similarities when compared to the SHR strain (Johnson et al., 1992; St Lezin et al., 1992).

Recently, our group has reported that the SHR strain, when compared to WRs, presents many behavioral changes that are specifically reversed by antipsychotic drugs and potentiated by proschizophrenia manipulations. Particularly, this strain displays impaired social interaction (mimics negative symptoms) that is specifically ameliorated by atypical antipsychotics and aggravated by amphetamine (Calzavara et al., 2011), hyperlocomotion (mimics positive symptoms) attenuated by antipsychotics and potentiated by amphetamine (Calzavara et al., 2011) and a deficit in contextual fear conditioning (CFC – model of emotional processing) that is reversed specifically by antipsychotics and potentiated by psychostimulants or other proschizophrenia manipulations, such as ketamine administration and sleep deprivation (Calzavara et al., 2009). Moreover, this strain has a basal PPI deficit specifically reverted by the atypical antipsychotic clozapine (Levin et al., 2011). These findings reinforce the SHR strain as an animal model to study several aspects of schizophrenia, including abnormalities in sensorimotor gating.

It is noteworthy that previous studies describe controversial results in relation to PPI in SHRs using control strains other than the WRs. Some studies show that SHRs present PPI deficits when compared to WKY (Ferguson and Cada, 2004; Kinkead et al., 2006), to Sprague-Dawley (SD; Ferguson and Cada, 2004), or to Lewis rats (Vendruscolo et al., 2006). Conversely, other studies demonstrate that PPI tended to be higher in SHRs and WKY than in SD rats (van den Buuse, 2004), or that SHRs have intermediate PPI values (Brown-Norway < SHR < SD < WKY – Palmer et al., 2000).

Several clinical and neurobiological findings suggest that some cannabinoids and the endocannabinoid system may be implicated in schizophrenia (Leweke et al., 2004; D’Souza et al., 2009). Some studies suggest that cannabis abuse is a method of self-medication for negative symptoms of the disease (Peralta and Cuesta, 1992; Bersani et al., 2002), or side effects of antipsychotics (Krystal et al., 1999; Verdoux et al., 2005). Other studies report that cannabis consumption may induce a psychotic state in normal individuals, worsen psychotic symptoms of schizophrenia patients, and facilitate precipitation of schizophrenia in vulnerable individuals (Ujike and Morita, 2004; Sewell et al., 2010). In *postmortem* studies, schizophrenia patients showed an increased density of the cannabinoid CB_1_ receptor binding in corticolimbic regions involved in this disorder (Dean et al., 2001; Zavitsanou et al., 2004; Newell et al., 2006; Dalton et al., 2011), indicating their role in negative symptoms and cognitive impairments (Gallinat et al., 2012). Moreover, elevated anandamide levels in the cerebrospinal fluid (Leweke et al., 2007; Koethe et al., 2009) and plasma (De Marchi et al., 2003) of patients with schizophrenia have been described.

Recently, we have demonstrated that CBD – a non-psychotomimetic compound of the *Cannabis sativa* plant that presents antipsychotic properties (Zuardi et al., 2012) – and rimonabant – a CB_1_ receptor antagonist (Rinaldi-Carmona et al., 1994) – were able to reverse the deficit in CFC task presented by SHRs. These results suggest that these drugs could constitute an alternative for the treatment of abnormalities in emotional context processing related to schizophrenia (Levin et al., 2012).

In order to further investigate the potential of the endocannabinoid system as target for the treatment of schizophrenia, the aim of this study was to evaluate the effects of cannabinoid drugs on the deficit of PPI presented by the SHR strain. For this purpose, dose–response curves of the following drugs were investigated: WIN55212,2 (cannabinoid agonist), rimonabant (CB_1_ antagonist), AM404 (anandamide uptake inhibitor), and CBD (indirect CB_1_/CB_2_ receptor antagonist, among other effects).

## MATERIALS AND METHODS

### ANIMALS

Five-month-old, male WRs and SHRs of our own colony were housed under conditions of controlled temperature (22–23°C) and lighting (12/12 h light/dark cycle, lights on at 07:00 am). Groups of five animals were kept in Plexiglas cages (41 cm × 34 cm × 16.5 cm), with free access to food and water. The animals were maintained in accordance with the guidelines of the Committee on Care and Use of Laboratory Animal Resources, National Research Council, USA. This study was approved by the Ethical Committee of Federal University of Sao Paulo. All rats used were drug-naive before each experiment.

### DRUGS

WIN55212,2 (Tocris) and CBD (THC-Pharm, Frankfurt, Germany and STI-Pharm, Brentwood, UK) were dissolved in Tween 80 and 0.9% saline. Rimonabant (Sanofi-Aventis^®^) was dissolved in ethanol, Tween 80 and 0.9% saline (ratio 1:1:18). AM404 (Tocris) was dissolved in Dimethyl Sulfoxide (DMSO) and Tween 80 and then diluted in 0.9% saline. Control solutions consisted of saline plus Tween 80, DMSO or ethanol, depending on the drugs used in each experiment. All drug solutions were injected intraperitoneally (i.p.) in a volume of 1 ml/kg body weight.

### APPARATUS

The rats were placed in a stabilimeter, which consisted of a wire-mesh cage (16.5 cm × 5.1 cm × 7.6 cm) suspended within a polyvinyl chloride frame (25 cm × 9 cm × 9 cm) attached to the response platform with four thumbnail-screws. The stabilimeter and platform were located inside a ventilated plywood sound attenuating chamber (64 cm × 60 cm × 40 cm). The floor of the stabilimiter consisted of six stainless steel bars 3.0 mm in diameter and spaced 1.5 cm apart. The startle reaction of the rats generated a pressure on the response platform and analog signals were amplified, digitized, and analyzed by software of the startle measure system (Insight, São Paulo, Brazil), that also controlled other parameters of the session (intensity of the acoustic stimulus, inter-stimulus interval, etc). Two loudspeakers located 10 cm above the floor, on each lateral side of the acoustic isolation chamber, were used to deliver the prepulse stimulus, the acoustic startle stimulus, and continuous background noise. Calibration procedures were conducted before the experiments to ensure equivalent sensitivities of the response platforms over the test period.

### PPI TESTING

The PPI testing began 30 min after the injection, by placing each animal in the stabilimeter cage where they were exposed to a background (65 dB) noise for 5 min. After this acclimatization period, the rats were submitted to a series of 10 stimuli (pulse alone – 120 dB, 50 ms duration), with an average inter-trial interval of 20 s. The purpose of this phase was to allow within-session habituation (not calculated herein) to the startle stimulus, and was not included in the calculation of PPI values nor of acoustic startle response (ASR). Thereafter, the PPI modulation of the acoustic startle was tested: this phase consisted of pseudorandomly delivered trials divided into four different categories presented with an average inter-trial interval of 20 s: 20 presentations of pulse alone (120 dB, 50 ms duration), 8 presentations of each prepulse alone (70, 75, and 80 dB, 3000 Hz frequency, 20 ms duration), 10 presentations of each prepulse + pulse (with 100 ms interval), and 8 no-stimulus trials (stabilimeter recordings obtained when no stimulus was presented). Mean amplitude of startle responses to pulse-alone (P) and prepulse-pulse (PP + P) trials was calculated for each subject. The level of PPI in each rat was determined by expressing the prepulse + pulse startle amplitude as a percentage decrease from pulse-alone startle amplitude, according to the following formula: %PPI = 100 - [100 × (PP/P)]. The ASR was expressed as the average of the 20 P trials.

All rats were submitted to a previous PPI session without drug administration. After this session, called “matching” (Swerdlow et al., 2005), rats were distributed into pharmacological groups [vehicle (VEH) or drug, for each experiment] matched for basal %PPI. Seven days later, each rat was submitted to a test session.

### STATISTICAL ANALYSIS

The ASR results were analyzed by two-way ANOVA (strain X treatment). The %PPI data were analyzed by three-way ANOVA with treatment and strain as between-subjects factors and prepulse intensity as within-subject factor. Since no interaction between strain and prepulse intensity, or treatment and prepulse intensity were detected, the *post hoc* comparison was then performed with the mean %PPI for the three prepulse intensities. Moreover, when an interaction between treatment and strain were detected, the data from each strain was analyzed separately. All *post hoc* comparisons were performed using Dunnett’s test, with VEH treatment as the control condition.

It is known that ASR might influence %PPI (Csomor et al., 2008). In this sense, the %PPI results were also analyzed by three-way repeated measures analysis of covariance (ANCOVA) ANCOVA, NMDA. with ASR as covariant (as suggested by Csomor et al., 2008), treatment and strain as between-subjects factors and prepulse intensity as within-subject factor. If a difference detected on ANOVA remained significant on ANCOVA, it is possible to state that the difference was not solely due to influences of ASR. The *p* < 0.05 was used as criterion for statistical significance. All statistical analyses were conducted on the software SPSS 20.

### EXPERIMENTAL DESIGN

#### Experiment 1: effect of WIN55212,2 (cannabinoid agonist) on %PPI and ASR of WRs and SHRs

Wistar rats and SHRs were treated with VEH, 0.3, 1, or 3 mg/kg WIN 55212,2 (WIN; *n* = 10, per strain and treatment). Thirty minutes later, the rats were submitted to the PPI test.

#### Experiment 2: effect of rimonabant (CB_1_antagonist) on %PPI and ASR of WRs and SHRs

Wistar rats and SHRs were treated with VEH or 0.75, 1.5, or 3 mg/kg rimonabant (RIMO; *n* = 9–11, per strain and treatment). Thirty minutes later, the animals were submitted to the PPI test.

#### Experiment 3: effect of AM404 (anandamide uptake inhibitor) on %PPI and ASR of WRs and SHRs

Wistar rats and SHRs were treated with VEH or 1, 5, or 10 mg/kg AM404 (AM; *n* = 9–11, per strain and treatment). Thirty minutes later, the animals were submitted to the PPI test.

#### Experiment 4: effect of cannabidiol (a cannabinoid with antipsychotic property) on %PPI and ASR of WRs and SHRs

Wistar rats and SHRs were treated with VEH or 15, 30, or 60 mg/kg CBD (*n* = 9–10, per strain and treatment). Thirty minutes later, the animals were submitted to the PPI test. In all the experiments, each animal was used for only one drug condition.

## RESULTS

### EXPERIMENT 1: EFFECT OF WIN 55212,2 (CANNABINOID AGONIST) ON %PPI AND ASR OF WRs AND SHRs

Two-way ANOVA showed only a significant effect of strain on ASR [*F*(1,72) = 31.93; *p *< 0.001]. WRs presented a higher ASR when compared to SHRs (**Table 1**).

**Table 1:** Acoustic startle response (ASR) of Wistar rats (WRs) and spontaneously hypertensive rats (SHRs) treated with vehicle (VEH), 0.3, 1, or 3 mg/kg WIN55212,2 (WIN – Experiment 1); VEH, 0.75, 1.5, or 3 mg/kg rimonabant (RIMO – Experiment 2); VEH, 1, 5, or 10 mg/kg AM404 (AM – Experiment 3); VEH, 15, 30, or 60 mg/kg cannabidiol (CBD – Experiment 4).

*Experiment 1*	VEH	WIN 0.3	WIN 1	WIN 3
WRs	464.6 ± 107.3 (*n* = 10)	422.3 ± 161.4 (*n* = 10)	588.3 ± 151.4 (*n* = 10)	588 ± 176.9 (*n* = 10)
SHRs	87.8 ± 10.7* (*n* = 10)	82.3 ± 10.7* (*n* = 10)	87.6 ± 15.1* (*n* = 10)	82.5 ± 27.3* (*n* = 10)
***Experiment 2***	**VEH**	**RIMO 0.75**	**RIMO 1.5**	**RIMO 3**
WRs	491.2 ± 109.3 (*n* = 11)	477.3 ± 138.8 (*n* = 11)	534.4 ± 144.1 (*n* = 11)	158.6 ± 76.3 (*n* = 11)
SHRs	198.7 ± 115.1* (*n* = 9)	71.1 ± 9.4* (*n* = 10)	140.5 ± 31.1* (*n* = 11)	85.2 ± 8.6* (*n* = 10)
***Experiment 3***	**VEH**	**AM 1**	**AM 5**	**AM 10**
WRs	478.0 ± 136.3 (*n* = 10)	539.6 ± 157.0 (*n* = 9)	613.2 ± 126.9 (*n* = 10)	450.7 ± 125.8 (*n* = 10)
SHRs	127.2 ± 13.8* (*n* = 10)	135.3 ± 27.9* (*n* = 11)	76.0 ± 10.6* (*n* = 10)	100.0 ± 23.2* (*n* = 10)
***Experiment 4***	**VEH**	**CBD 15**	**CBD 30**	**CBD 60**
WRs	486.2 ± 188.3 (*n* = 10)	239.6 ± 98.2 (*n* = 9)	234.3 ± 56.6 (*n* = 10)	343.7 ± 72.3 (*n* = 10)
SHRs	92.5 ± 8.2* (*n* = 10)	76.1 ± 8.5* (*n* = 10)	117.4 ± 13.6* (*n* = 10)	57.1 ± 6.9* (*n* = 10)

*p < 0.001 compared to WRs. Two-way ANOVA. Data are reported as mean ± SE.

**FIGURE 1:**
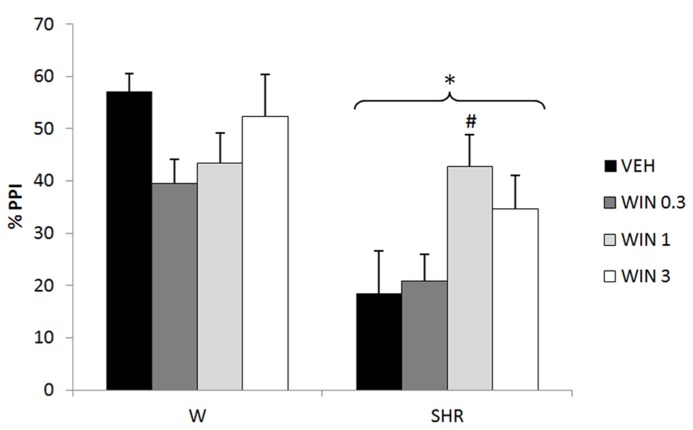
**%PPI of Wistar rats (WRs) and spontaneously hypertensive rats (SHRs) treated with vehicle (VEH), 0.3, 1, or 3 mg/kg WIN55212,2 (WIN).** *p < 0.001 compared to WRs. #*p* < 0.05 compared to VEH group of the same strain. Three-way repeated measures ANOVA followed by Dunnett’s test. Data are reported as mean ± SE.

Three-way repeated measures ANOVA revealed significant effects of prepulse intensity (reflecting that the more intense the prepulse, the higher the PPI) [*F*(2,144) = 26.65; *p* < 0.001], strain (reflecting a decreased PPI in SHRs) [*F*(1,72) = 18.87; *p* < 0.001], and an interaction between strain and treatment [*F*(3,72) = 3.15, *p* = 0.030] on %PPI. Neither treatment nor interactions between %PPI and treatment or strain reached significance. All the effects detected on ANOVA remained significant on ANCOVA. *Post hoc* analysis showed that treatment with 1 mg/kg WIN increased % PPI in SHRs (*p* = 0.035; **Figure 1**).

### EXPERIMENT 2: EFFECT OF RIMONABANT (CB_1_ ANTAGONIST) ON %PPI AND ASR OF WRs AND SHRs

Two-way ANOVA showed only a significant effect of strain on ASR [*F*(1,75) = 19.71; *p* < 0.001]. WRs presented a higher ASR when compared to SHRs (**Table 1**).

**FIGURE 2:**
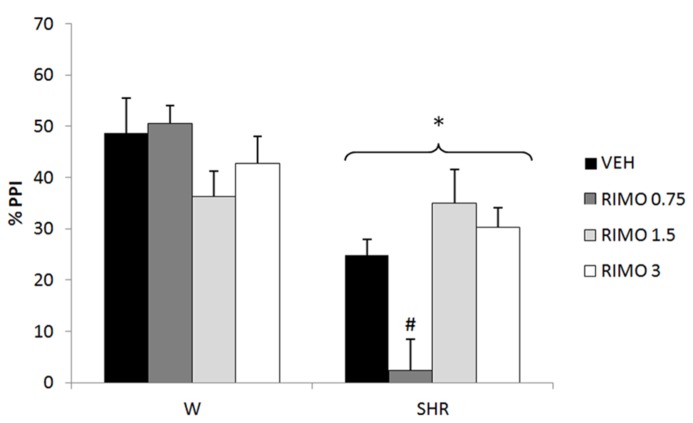
**%PPI of Wistar rats (WRs) and spontaneously hypertensive rats (SHRs) treated with vehicle (VEH), 0.75, 1.5, or 3 mg/kg rimonabant (RIMO).** *p < 0.001 compared to WRs. #p < 0.05 compared to VEH group of the same strain. Three-way repeated measures ANOVA followed by Dunnett’s test. Data are reported as mean ± SE.

Three-way repeated measures ANOVA showed significant effects of prepulse intensity (reflecting that the more intense the prepulse, the higher the PPI) [*F*(2,150) = 53.45; *p* < 0.001], strain (reflecting a decreased PPI in SHRs) [*F*(1,75) = 32.50; *p* < 0.001] and an interaction between strain and treatment [*F*(3,75) = 7.26, *p* < 0.001] on %PPI. Neither treatment nor interactions between %PPI and treatment or strain reached significance. All the effects detected on ANOVA remained significant on ANCOVA. *Post hoc* analysis showed that that treatment with 0.75 mg/kg RIMO decreased %PPI in SHRs (*p* = 0.017; **Figure 2**).

### EXPERIMENT 3: EFFECT OF AM404 (ANANDAMIDE UPTAKE INHIBITOR) ON %PPI AND ASR OF WRs AND SHRs

Two-way ANOVA showed only a significant effect of strain on ASR [*F*(1,72) = 37.48; *p* < 0.001]. WRs presented a higher ASR when compared to SHRs (**Table 1**).

**FIGURE 3:**
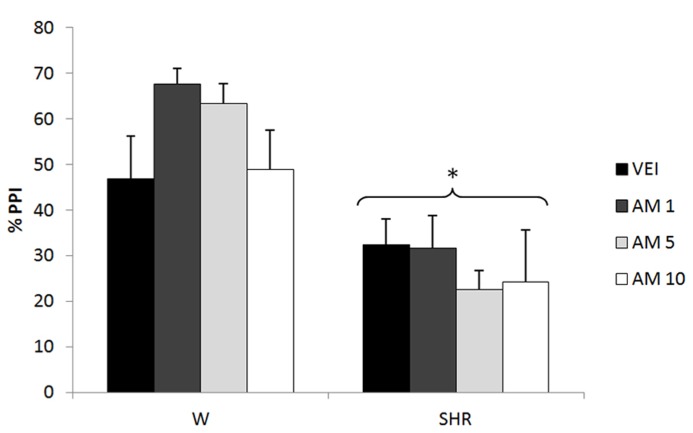
**%PPI of Wistar rats (WRs) and spontaneously hypertensive rats (SHRs) treated with vehicle (VEH), 1, 5, or 10 mg/kg AM404 (AM).** *p < 0.001 compared to WRs. Three-way repeated measures ANOVA followed by Dunnett’s test. Data are reported as mean ± SE.

Three-way repeated measures ANOVA showed significant effects of prepulse intensity (reflecting increased PPI by increasing the intensity of prepulse) [*F*(2,144) = 10,89; *p* < 0.001] and strain (reflecting a decreased PPI in SHRs) [*F*(1,72) = 31.23; *p* < 0.001]. Neither treatment nor any possible interaction with this factor reached significance (reflecting that there was no effect of AM404 on %PPI in any of the strains). All the effects detected on ANOVA remained significant on ANCOVA. (**Figure 3**).

### EXPERIMENT 4: EFFECT OF CANNABIDIOL (A CANNABINOID WITH ANTIPSYCHOTIC PROPERTY) ON %PPI AND ASR OF WRs AND SHRs

Two-way ANOVA showed only a significant effect of strain on ASR [*F*(1,71) = 17.27; *p* < 0.001]. WRs presented a higher ASR when compared to SHRs (**Table 1**).

**FIGURE 4:**
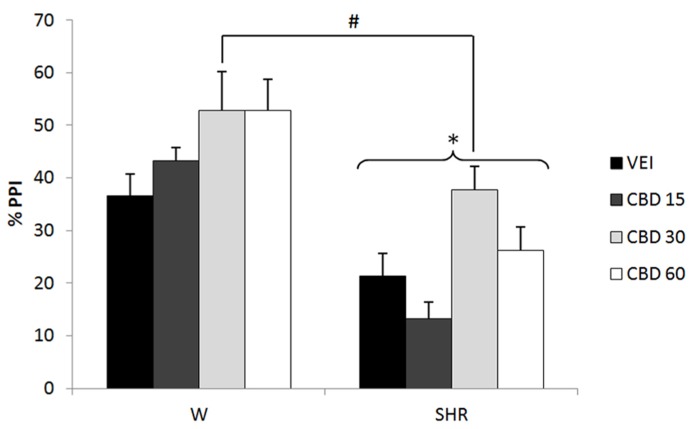
**%PPI of Wistar rats (WRs) and spontaneously hypertensive rats (SHRs) treated with vehicle (VEH) or 15, 30, or 60 mg/kg cannabidiol (CBD).** *p < 0.001 compared to WRs. #*p* < 0.05 compared to VEH group. Three-way repeated measures ANOVA followed by Dunnett’s test. Data are reported as mean ± SE.

Three-way repeated measures ANOVA showed significant effects of prepulse intensity (reflecting increased PPI by increasing the intensity of prepulse) [*F*(2,142) = 13.83; *p* < 0.001], strain (reflecting a decreased PPI in SHRs) [*F*(1,71) = 41.08; *p* < 0.001] and treatment [*F*(3,71) = 5.99; *p* = 0.001]. None of the interactions between these factors reached significance. All the effects detected on ANOVA remained significant on ANCOVA. *Post hoc* analysis revealed that treatment with 30 mg/kg CBD increased PPI response (*p* = 0.020; **Figure 4**).

## DISCUSSION

Our data show that SHRs have deficits in baseline PPI (corroborating previous data from our group – Levin et al., 2011) and ASR when compared to WRs. These findings are in accordance with clinical studies in schizophrenia which show disrupted PPI (Braff and Geyer, 1990; Braff et al., 1992, 1999; Weike et al., 2000), and lower startle reactivity (Quednow et al., 2006, 2008 – but see Grillon et al., 1992; Weike et al., 2000; Xue et al., 2012 and comments on the possible reasons for these negative results in Quednow et al., 2006) when compared to controls, and reinforce the SHR strain as an animal model to study abnormalities in sensorimotor gating related to schizophrenia.

It could be argued that the deficit in PPI in SHR might be a consequence of the lower level of ASR displayed by this strain, observed also in other studies (using WKY and SD rats as control strains – Ferguson and Cada, 2003; van den Buuse, 2004). Nevertheless, this does not seem to be the case because the changes in PPI induced by the drugs tested (increase or decrease – described below) were not accompanied by changes in ASR levels (**Table 1**). In addition, previous work in humans and rodents suggests that lower baseline ASR is associated with higher PPI (Csomor et al., 2008), which is opposite to what we observed in SHR. Therefore, the reduced PPI in SHR is probably not due to their diminished ASR. However, since ASR might influence PPI and the drugs used could have induced subtle changes in ASR, the data were re-analyzed with ASR as covariate (as suggested by Csomor et al., 2008). The results of these analyses reinforce that the effects seen on %PPI were not due to differences in ASR.

As commented above, several clinical and neurobiological findings suggest that cannabinoids and the endocannabinoid system are implicated in schizophrenia (Leweke et al., 2004; D’Souza et al., 2009). Our data show that cannabinoid drugs differentially modulate the spontaneous deficit of PPI presented by SHRs.

Concerning cannabinoid agonists, the interaction of Δ^9^- tetrahydrocannabidiol (THC – the primary psychotropic constituent of *Cannabis sativa* plant) with CB_1_ receptors seems to be responsible for its psychotomimetic effects: induction of a psychotic state in normal individuals, worsening of psychotic symptoms of schizophrenic patients, and precipitation of schizophrenia in vulnerable individuals (Ujike and Morita, 2004; Sewell et al., 2010). Concerning specifically PPI, although one clinical study did not observe alterations of PPI in drug-free chronic cannabis users (Quednow et al., 2004), another showed that chronic cannabis use in healthy individuals was associated with attention-modulated reduction in PPI resembling the PPI deficit in schizophrenia (Kedzior and Martin-Iverson, 2006). In addition, Mathias et al. (2011) did not observe differences in PPI among adolescent cannabis users and controls, but they detected a more rapid decline in PPI in frequent cannabis users. The authors suggested that this could reflect a progressive reduction in the quality of information processing or sustained attention across the PPI session.

With respect to rodent studies, treatment with the cannabinoid agonist WIN has been reported to disrupt sensorimotor gating in systemically treated animals (Schneider and Koch, 2002; Wegener et al., 2008), and after intra-prefrontal cortex and intra-ventral hippocampus infusion (Wegener et al., 2008). It also impaired recognition memory (Schneider and Koch, 2002), CFC (Pamplona and Takahashi, 2006) and social interaction (Almeida et al., in press) in rats. Consistent with this, our results showed a trend toward a decrease in PPI in WRs treated with the lowest dose of this compound (*p* = 0.095 – **Figure 1**). On the other hand, in accordance the ability of WIN (1 mg/kg) to reverse the basal PPI deficit in SHRs (**Figure 1**), other studies have shown that in animals with low basal PPI, such as phencyclidine-treated rats (which induces “schizophrenia-like behaviors” – Gouzoulis-Mayfrank et al., 2005) and psychosocially stressed mice (Brzozka et al., 2011), treatment with WIN reverses this deficit. In addition, other behavioral abnormalities induced by phencyclidine are also reversed by this compound: impairments in novel object recognition, and in social interaction (Spano et al., 2010), as well as hyperlocomotion and anxiogenic behavior (Spano et al., 2012). Consistent with this, another study from our group demonstrates a beneficial effect of WIN on the deficit of social interaction (mimicking the negative symptoms of schizophrenia – Sams-Dodd, 1998) presented by SHRs (Almeida et al., in press). Of note, the impairment of social interaction of SHRs was attenuated only by atypical antipsychotics (Calzavara et al., 2011). Finally, a study in a small group of schizophrenia patients reported that treatment with synthetic THC (dronabinol) improves the symptoms of the disease (Schwarcz et al., 2009).

Taken as a whole, these data indicate that cannabinoid agonists may present differential effects in controls and schizophrenia. This observation might reflect dysfunctions of the endocannabinoid system associated with schizophrenia that would also be displayed by SHRs. In this sense, recently, our group showed that, as observed in schizophrenia patients (Dean et al., 2001; Zavitsanou et al., 2004; Dalton et al., 2011), the SHR strain has a higher CB_1_ receptor density in the prefrontal and anterior cingulate cortices when compared to WRs (Levin et al., submitted).

Only one dose of WIN increased PPI in SHR. Several previous studies have demonstrated similar biphasic effects of cannabinoid agonists in different paradigms: low doses of these components usually induce anxiolytic-like effects, while higher doses are anxiogenic or ineffective (Hill and Gorzalka, 2004; Viveros et al., 2005; Fogaca et al., 2012). Moreover, a low dose (0.1 mg/kg) of WIN stimulated motor activity, whereas a higher dose (1 mg/kg) decreased this response (Polissidis et al., 2013). Similarly, in mice submitted to a CFC task, THC exerted biphasic effects on fear-coping strategies, with lower and higher doses favoring active and passive responses, respectively (Metna-Laurent et al., 2012). This profile was also detected in clinical studies which observed that low and moderate THC doses had anxiolytic and euphoric properties, while higher doses produced anxiogenic responses (for review, see Crippa et al., 2010). These variable effects, depending on the dose, could be due to the wide neuroanatomical distribution of the endocannabinoid system and its modulatory effects on both GABAergic and glutamatergic neurons (Moreira and Lutz, 2008; Fogaca et al., 2012; Metna-Laurent et al., 2012). This could explain the inverted U-shaped dose–response curve of WIN on PPI in SHRs (**Figure 1**), indicating that this dose-dependent effect of cannabinoid agonists can also be seen for sensorimotor gating deficits.

Regarding the effects of the CB_1_ receptor antagonist rimonabant, our data revealed that the lowest dose (0.75 mg/kg) worsened the PPI deficit presented by SHRs (**Figure 2**). On the other hand, a recent study of our group showed that 3 mg/kg rimonabant was able to reverse the deficit in CFC in SHRs (Levin et al., 2012). Any of the doses tested was able to modify the impairment in social interaction and hyperlocomotion presented by this strain (Almeida et al., in press). In this sense, the effects of CB_1_ antagonist seem to depend on the behavior evaluated and the dose used.

Indeed, previous studies on the effects of rimonabant on schizophrenia-like behaviors in animal models have shown contrasting results (Roser et al., 2010). While some studies showed that rimonabant was able to counteract the disruption of PPI produced by the N-methyl-D-aspartate (NMDA) antagonists, phencyclidine and Dizocilpine (MK-801), and by the dopamine agonist, apomorphine (Malone et al., 2004; Ballmaier et al., 2007), others demonstrated that this drug did not reverse the PPI-disruptive effects of apomorphine, amphetamine or MK-801, nor the amphetamine-induced hyperactivity or stereotypy in rats (Martin et al., 2003). Malone and Taylor (2006) demonstrated that rimonabant reversed the THC-induced deficits in PPI in socially isolated rats (a long-term environmental manipulation used as an animal model of schizophrenia – Weiss et al., 2000) but did not reverse the isolation-induced deficits in PPI *per se* (Malone and Taylor, 2006). On the other hand, Ferrer et al. (2007) showed that rimonabant potentiated stereotyped behavior induced by the D_1_ and D_2_ dopamine agonists, (±)-1-phenyl-2,3,4,5-tetrahydro-(1H)-3-benzazepine-7,8-diol (SKF-38393) and quinpirole (model of positive symptoms – Ferrer et al., 2007). Finally, rimonabant increased c-fos expression in mesocorticolimbic areas of rats (Alonso et al., 1999), similar to typical and atypical antipsychotics (Robertson and Fibiger, 1992). In addition, other CB_1_ antagonists (AM251 and AVE 1625) seem to reverse the cognitive deficits observed in pharmacological (Black et al., 2011; Guidali et al., 2011) and neurodevelopmental (Zamberletti et al., 2012) animal models of schizophrenia.

Interestingly, no effect was observed in WRs corroborating previous data that show that rimonabant is not able to modify PPI under normal conditions (Martin et al., 2003; Ballmaier et al., 2007), and reinforcing its specificity to “schizophrenia-like” behaviors.

Clinical data with rimonabant are also conflicting. Some clinical trials failed to show any antipsychotic effect of rimonabant (Meltzer et al., 2004). On the other hand, in a small sample-size study, rimonabant produced a significant improvement in Brief Psychiatric Rating Scale (BPRS) of schizophrenic patients (Kelly et al., 2011). Conversely, Roser et al. (2011) showed that rimonabant produced a significant deficit in auditory sensory memory in the ketamine model of schizophrenia.

Taken together, under our experimental conditions, the cannabinoid receptor agonist WIN reversed PPI deficits in SHRs, whereas the CB_1_ antagonist rimonabant enhanced this deficit, indicating that the CB_1_ receptor might be involved in both basal PPI deficits seen here as well as in the modulatory effects of these drugs. High densities of CB_1_ receptors have been found in brain areas that regulate sensorimotor gating such as prefrontal cortex, amygdala and hippocampus (Dissanayake et al., 2013). Moreover, CB_1_ receptors have a modulatory role on specific neurotransmitter systems, mainly glutamate, GABA and dopamine (Schlicker and Kathmann, 2001), which have a critical role in the PPI processing.

Supporting the involvement of the endocannabinoid system in schizophrenia, elevated anandamide levels in the cerebrospinal fluid (Leweke et al., 1999; Leweke et al., 2007; Koethe et al., 2009) and plasma (De Marchi et al., 2003) of patients with schizophrenia have been described. Moreover, studies showed that anandamide levels were inversely correlated with psychotic symptoms (Giuffrida et al., 2004) and low levels of this endocannabinoid are a risk factor for psychosis (Koethe et al., 2009). These studies suggest that increased levels of anandamide in schizophrenia might play a protective role to counteract the abnormalities in neurotransmission during acute symptoms. Reinforcing this, Leweke et al. (2012) showed that treatment with CBD resulted in a significant increase in anandamide levels, which was accompanied by clinical improvement in schizophrenic patients.

In accordance with a protective role of anandamide, 5 mg/kg AM404 (anandamide uptake inhibitor) was able to attenuate the hyperlocomotion and impaired social interaction presented by SHR (Almeida et al., in press). It is noteworthy that the same range of doses used in that study and in the present one, was previously shown to increase anandamide levels in plasma (Giuffrida et al., 2000) and brain regions (Bortolato et al., 2006) of rats. Local injections of this compound also prevented the stereotypy and hyperlocomotion induced by dopamine receptor agonist treatment (Beltramo et al., 2000). Accordingly, Seillier et al. (2010) demonstrated that fatty acid amidrohydrolase inhibitors reversed PCP-induced social impairment. Nevertheless, in the present study AM404 did not modify the PPI deficit in SHRs at any dose (**Figure 3**). In this sense, the possible antipsychotic profile of anandamide enhancers does not seem to include all the “schizophrenia-like” behaviors.

In control WRs, AM404 did not modify PPI (**Figure 3**). In contrast, a previous study suggested that AM404 (at similar doses used in the present study) disrupted PPI (Fernandez-Espejo and Galan-Rodriguez, 2004), increased locomotion and decreased social interaction (Almeida et al., in press) in control strains. Differences in the behavioral paradigms or rat strains might account for the variable profile of AM404 in control animals.

Cannabidiol, one of the major constituent of *Cannabis sativa* (Grlie, 1976), is devoid of the typical psychotomimetic effects of the plant (Zuardi, 2008; Crippa et al., 2010; Zuardi et al., 2010). Several clinical studies revealed that this component does induce central effects (Zuardi et al., 2010) including antipsychotic properties (Zuardi et al., 2006, 2010, 2012; Leweke et al., 2007). Several clinical trials reveal that antipsychotic-like activity of CBD can be demonstrated against psychotic symptoms induced in healthy volunteers (Leweke et al., 2000; Zuardi et al., 2006; Borgwardt et al., 2008; Bhattacharyya et al., 2009, 2010; Winton-Brown et al., 2011), and in patients with Parkinson’s Disease (Zuardi et al., 2009) or schizophrenia (Zuardi et al., 1995, 2006; Leweke et al., 2007, 2012). Interestingly, these studies showed that CBD produces fewer (Leweke et al., 2007) or no (Zuardi et al., 1995, 2006, 2009; Leweke et al., 2012) side effects when compared to other antipsychotics and suggest it may be an effective and well-tolerated alternative treatment for schizophrenia (Zuardi,2008; Zuardi et al., 2012; Bergamaschi et al., 2011).

This profile is supported by several animal studies (Zuardi et al., 2006; Roser and Haussleiter, 2012). CBD was able to reverse MK-801-induced disruption of PPI (Long et al., 2006), inhibited the hyperlocomotion induced by amphetamine and ketamine in mice (Moreira and Guimaraes, 2005), and reversed the reduction in social interaction produced by THC (Malone et al., 2009) and MK-801 (Gururajan et al., 2011) in rats. Long et al. (2012) showed that long-term CBD enhanced social interaction in neuregulin-1 mutant mice (a putative animal model of schizophrenia – Long et al., 2012). Recently, our group showed that CBD was able to reverse the deficit in CFC presented by SHRs (Levin et al., 2012). Moreover, similar to the atypical antipsychotic clozapine (Robertson and Fibiger, 1992), CBD induced c-fos immunoreactivity in the nucleus accumbens (but not in the striatum) of rats (Guimaraes et al., 2004). The present results further support the antipsychotic profile of CBD since the dose of 30 mg/kg was able to reverse the deficit in PPI presented by SHRs.

Interestingly, contrary to the specificity of effect for PPI deficits in SHRs seen with WIN and rimonabant, the same dose of CBD increased PPI in both WRs and SHRs (**Figure 4**). In this respect, it might be suggested that the effects of CBD on molecular targets other than cannabinoid receptors (affected by WIN and rimonabant) could account for its different profile of action. The molecular targets of CBD are not completely elucidated. Studies have suggested that CBD activates vaniloid receptors transient receptor potential cation channel subfamily V member 1 (TRPV1), inhibits the cellular uptake and hydrolysis of anandamide (Bisogno et al., 2001), acts as an agonist at the 5HT_1A_ receptor (Russo et al., 2005), and acts as an indirect CB_1_/CB_2_ antagonist (Pertwee, 2008), as well as an antagonist at the novel cannabinoid receptor G protein-coupled receptor 55 (Campos et al., 2012). Although it was not the aim of this study to reveal the neural mechanism behind the effects of cannabinoid drugs on PPI, it is interesting to note that the clinical improvement in schizophrenic patients induced by CBD was accompanied by an increase in anandamide levels (Leweke et al., 2012). However, our data suggest that the enhancing effect of CBD on PPI does not seem to be due only to an increase in anandamide levels, since AM404 (anandamide uptake inhibitor) did not modify this response. In this context, Bisogno et al. (2001) revealed that CBD is more potent in activating TRPV1 receptors than in inhibiting anandamide hydrolysis and uptake. Strengthening the role of TRPV1 in the beneficial effect of CBD, the attenuation of MK-801-induced PPI deficits is prevented by pretreatment with capsazepine, a TRPV1 antagonist (Long et al., 2006).

In conclusion, our results indicate that the sensorimotor gating impairment in SHRs can be modulated by cannabinoid drugs pointing to these compounds as potential therapeutic strategies. More specifically, the present study suggests a beneficial property of a direct cannabinoid receptor agonist (WIN55,212) and of CBD on the PPI deficits associated to schizophrenia.

## AUTHOR CONTRIBUTIONS

Raquel Levin, Antonio W. Zuardi, Jaime E. C. Hallak, José Alexandre S. Crippa, and Vanessa C. Abílio designed the study. Authors Raquel Levin, Fernanda F. Peres, Valéria Almeida, and Mariana B. Calzavara conducted the experiments, statistical analyses and managed the literature search and analyses. Authors Raquel Levin and Vanessa C. Abílio wrote the first draft of the manuscript. All authors contributed to and have approved the final manuscript.

## Conflict of Interest Statement

The authors declare that the research was conducted in the absence of any commercial or financial relationships that could be construed as a potential conflict of interest.
